# A Review of the Use of Virtual Reality for Teaching Radiology in Conjunction With Anatomy

**DOI:** 10.7759/cureus.20174

**Published:** 2021-12-05

**Authors:** Dimitrios Chytas, Marios Salmas, Theano Demesticha, George Noussios, Georgios Paraskevas, Chrysanthos Chrysanthou, Irene Asouhidou, Anastasios Katsourakis, Aliki Fiska

**Affiliations:** 1 Anatomy, University of Peloponnese, Sparta, GRC; 2 Anatomy, National and Kapodistrian University of Athens, School of Medicine, Athens, GRC; 3 Physical Education and Sports Sciences, Aristotle University of Thessaloniki, Thessaloniki, GRC; 4 Anatomy and Surgical Anatomy, Aristotle University of Thessaloniki, Thessaloniki, GRC; 5 Surgery, General Hospital of Thessaloniki "O Agios Dimitrios", Thessaloniki, GRC; 6 Anatomy, Democritus University of Thrace, Alexandroupolis, GRC

**Keywords:** review, integration, radiology education, virtual reality, anatomy education

## Abstract

Incorporation of radiology into anatomy education is a frequently used teaching strategy. Our purpose was to investigate to what extent virtual reality can play a significant role when radiology is taught in conjunction with anatomy. PubMed, SCOPUS, Education Resources Information Center, and Cochrane databases were searched for articles with the aim to evaluate the outcomes of incorporation of radiology in anatomy education, using virtual reality. From each included paper, the following data were extracted: authors, number of participants, type of study (comparative or not), level of outcome according to Kirkpatrick hierarchy, and outcomes of the use of virtual reality when radiology was incorporated in anatomy education.

Seven papers were included. From them, three were comparative and evaluated students’ academic performance after the educational intervention, while four were non-comparative and evaluated only students’ opinions about the intervention. In all studies, the use of virtual reality for the incorporation of radiology into anatomy teaching was positively perceived. Also, the three studies which evaluated academic performance showed that virtual reality was effective in terms of enhancing anatomy knowledge.

The implementation of virtual reality for the incorporation of radiology into anatomy education has been accompanied by positive outcomes. These outcomes may encourage educators to teach radiology in conjunction with anatomy using virtual reality.

## Introduction and background

Incorporation of radiology into anatomy education is a common practice in medical curricula [1] and can be justified by the unsatisfactory anatomical knowledge of many senior medical students who enter radiology rotations [2]. Teaching radiology in conjunction with anatomy has been accompanied by encouraging learning outcomes [3]. These outcomes have been attributed to the fact that using radiologic images in anatomy teaching stimulates students’ interest, given that those images are the principal means via which doctors encounter a patient’s anatomy [3]. Thus, learning radiologic anatomy seems relevant to most medical students’ future clinical practice. Despite the suggestion for integration of radiology into anatomy teaching, there is currently a lack of reviews of evidence about the role of specific methods in this integration [3].

Virtual reality (VR) is a technology that either immerses the user in a digital environment via specific devices or simply comprises digital models presented on a two-dimensional (2D) computer screen [4]. The literature has demonstrated that this technology is an effective anatomy teaching modality [5]. Especially in the modern education era, “virtualization” has become an increasingly common practice, in particular during the coronavirus disease 2019 pandemic [6]. A remarkable characteristic of VR is that it is a versatile teaching method, being able to permit or not to interact with virtual models [7], as well as digital dissection [2]. It has been also proposed that VR is a “golden” opportunity for radiologists to provide clinical relevance to undergraduate anatomy education [2]. However, there is, to the best of our knowledge, a lack of review of the evidence about the value of VR in teaching radiology in conjunction with anatomy. Thus, the purpose of the current review was to investigate this value and explore to what extent VR can be considered as an effective means of incorporation of radiology into anatomy education.

## Review

Materials and methods

Four reviewers searched independently in PubMed, SCOPUS, Education Resources Information Center (ERIC), and Cochrane database with the following keywords: “radiology” OR “radiologic” OR “radiological” AND "anatomy" AND "education" OR "teaching" OR "learning" AND “virtual.” The search was completed on August 25, 2021. To be up-to-date, we did not expand our search to articles that were published before the last decade, so we searched for studies published after 2011. To find relevant papers which were not retrieved after the initial search, the list of references of each included paper was checked. The reviewers discussed any disagreement and, when it could not be resolved with discussion, it was planned that the senior author would decide.

Articles that met our inclusion criteria were those with a purpose to investigate the outcomes of the use of VR for teaching radiology in conjunction with anatomy, published in peer-reviewed journals and written in English. Articles that did not focus on the aforementioned use of VR, duplicates, reviews, letters to the editor, commentaries, and conference articles were excluded. Our screening process involved title, abstract and full text. If the title did not permit each reviewer to decide whether the paper would be included or excluded, the next stage was the screening of the abstract. If the abstract was inadequate again, each reviewer screened the full text. From each included article we extracted authors, type of study (comparative or not), number of participants, level of outcome according to Kirkpatrick hierarchy, and educational outcomes of the use of VR for teaching radiology in conjunction with anatomy (Table 1) [8,9].

**Table 1:** Kirkpatrick hierarchy (levels of training evaluation) Source: Kirkpatrick [8] and Hammick [9].

Kirkpatrick hierarchy		
Level 1	Reaction	Relates to participants’ opinions on the learning experience.
Level 2a	Change of attitudes-perceptions	Relates to changes in the participants’ attitudes or perceptions after the educational intervention.
Level 2b	Change of knowledge-skills	Relates to the acquisition of knowledge and skills after the educational intervention.
Level 3	Behavioral change	Relates to the change of behavior in the workplace due to the educational intervention.
Level 4a	Change in organizational practice	Significant changes in the delivery of care, due to an educational program.
Level 4b	Benefits to patients	Improvement of patients’ health due to an educational program.

Results

In total, 91 articles were initially identified. We excluded seven review articles, four commentaries, one conference article, one paper that concerned augmented reality, which is technologically different from VR, and 71 irrelevant articles. Thus, seven papers were included (Table 2) [10-16]. There were four articles that were non-comparative and evaluated only students’ opinions about the incorporation of radiology into anatomy education using VR, thus they had a level 1 in Kirkpatrick hierarchy [13-16]. There were three articles that were comparative and evaluated not only students’ opinions but also the results of their examination after the educational intervention, thus they had a level 2b in Kirkpatrick hierarchy (Table 2) [10-12]. In total, the participants were 898 medical students.

**Table 2:** Basic characteristics of the studies included in the review

Authors	Number of participants (medical students)	Kirkpatrick level	Type of study
Lorenzo-Alvarez et al. [10]	197	2b	Comparative
Paech et al. [11]	238	2b	Comparative
Rudolphi-Solero et al. [12]	52	2b	Comparative
Darras et al. [13]	202	1	Non-comparative
Rizvi and Borges [14]	18	1	Non-comparative
May et al. [15]	104	1	Non-comparative
Darras et al. [16]	87	1	Non-comparative

Comparative Studies

Lorenzo-Alvarez et al. included 107 medical students who received only traditional radiology teaching (via conferences, seminars, hospital practices, and access to multimedia contents) and 90 medical students who were additionally taught radiologic anatomy via a VR-based game [10]. The participants of the intervention group received radiologic anatomy teaching, and then answered questions that were asked after each session of the VR-based game. One month after the educational intervention, it was found that 34 students who participated in the VR-based game (37.8% of the group) performed significantly better in examinations than 41 students who did not participate (38.3% of the group). However, three months later, 82 students (91.1%) from the former group and 80 (74.8%) from the latter performed equally in their examinations. Seventy-seven (85.5%) participants of the VR-based game thought that this teaching method was effective, with an average grade of approximately four on a five-point Likert scale.

The paper by Paech et al. comprised 238 medical students, who received anatomy teaching and were divided into three groups, which belonged to three different academic years [11]. The training of the first group (50 participants) included radiologic image interpretation and access to cadaver computed tomography (CT) scans, visualized on a CT workstation and on life-size virtual dissection tables. The training of the second group (90 participants) comprised only radiologic image interpretation, while the education of the third group (98 participants; conventional anatomy group) did not include radiologic image interpretation. The first group performed significantly better in anatomy examinations in comparison with the other two groups. In addition, approximately two-thirds of the students thought that the incorporation of cadaveric CT scans and virtual dissection in the course led to better anatomy comprehension.

Rudolphi-Solero et al. included 52 medical students who were voluntarily taught radiologic anatomy, with the addition of a VR game-based approach to their curriculum [12]. The students were divided into 13 teams and attended a course of six weekly stages, concerning thoracic, abdominal, and musculoskeletal radiological anatomy and semiology. Thirty-five participants filled an evaluation questionnaire and rated their overall experience with a mean grade of 8.1 (in a 10-point Likert scale), while the educational usefulness of the intervention was rated with a mean grade of 8.7/10. A test was undertaken, one month after the intervention, by 45 participants and 83 non-participants. The former group achieved significantly higher scores than the latter. The final examination at the end of the academic year was undertaken by 49 participants and 96 non-participants. The former group scored again significantly better than the latter.

Non-Comparative Studies

Darras et al. included 202 medical students who were taught radiologic anatomy via virtual dissection tables, which were integrated into a cadaver-based anatomy course [13]. The virtual dissection was performed by a radiologist who demonstrated the normal anatomy on CT scans of living patients. In total, six virtual dissection tables were developed, representing heart, lungs and mediastinum, abdominal vessels, abdominal organs, spine, and bony pelvis. After the educational intervention, the participants completed a questionnaire and 78.7% of them stated that seeing the radiologic anatomy on the virtual dissection table reinforced their understanding of the cadaveric anatomy and that the content shown helped them understand the clinical applications of anatomy.

The study by Rizvi and Borges comprised 18 medical students who received radiologic anatomy teaching via a VR platform [14]. The virtual workstations included five dual-monitor desktops, while the course also comprised 25 representative cases each in the chest, abdomen, and pelvis (for a three-day body imaging rotation) and 50 cases each related to the brain, head and neck, and spine (for a three-day neuroradiology rotation). A PowerPoint presentation concerning the basic normal anatomy on plain x-rays, ultrasound, CT, and magnetic resonance imaging (MRI) was also carried out. Of the 18 participants, 12 strongly agreed and six agreed to be satisfied with the educational intervention. Fourteen students strongly agreed and four agreed that the course resulted in a better understanding of imaging procedures and recognition of basic anatomic structures.

May et al. developed an anatomy curriculum that comprised navigating inside the bodies of various living individuals utilizing a CT viewer and performing virtual dissection, with the aid of radiologists [15]. Each region of the body had its own folder on the CT portal, containing several cases concerning different aspects of the body. This anatomy curriculum was applied for three academic years, beginning from 2008 to 2009. The authors included 104 medical students in their article (54 from the academic year 2008-2009 and 50 from the academic year 2010-2011), who assessed the new curriculum after the end of the course. Most of the cohort of 2010-2011 argued that the new curriculum significantly contributed to their learning of anatomy (68%), while only 13% of the cohort of 2008-2009 had the same opinion.

The questionnaire study by Darras et al. comprised 87 medical students who received radiologic anatomy teaching via virtual dissection with clinical cases [16]. These cases (seven CT scans and one MRI scan) were selected from a virtual dissection table database and, then, groups of six to eight students performed virtual dissection tasks concerning four topics (spine, chest, abdomen, and pelvis) with a tutor, who was a radiologist. From the respondents, approximately 64% reported that the teaching intervention enhanced their understanding of visuospatial relationships, while 76% perceived that their radiologic anatomy knowledge was improved.

Discussion

All studies included in the current review showed that the incorporation of radiology into anatomy education using VR has led to positive results, concerning not only students’ opinions [10-16] but also their knowledge [10-12]. It can be noted that four out of the seven studies included in the review [11,13,15,16] investigated the use of virtual dissection, while three studies used VR platforms which did not give the opportunity for digital dissection [10,12,14]. Either with the performance of virtual dissection or not, the use of VR in radiological anatomy teaching has been proved favorable. The clinical relevance provided to anatomy by incorporation of radiology teaching may have contributed to this result, given that clinical orientation may increase students’ motivation to learn anatomy [17]. Also, when students can interact with the virtual models, the learning outcomes may be better in comparison with passive viewing and this hypothesis is a potential field of further research [7]. It seems that the implementation of VR in the integration of radiology into anatomy curricula should be encouraged and can be proved a useful tool to enhance medical students’ anatomy learning experience.

Impact on Examinations Results

Three studies explored the impact of VR on medical students’ knowledge about radiologic anatomy [10-12]. They demonstrated significant superiority of VR to other methods in terms of students’ examinations results. It can be noted that in the paper by Lorenzo-Alvarez et al., this superiority was only demonstrated one month after the educational intervention, while, three months after it, there was an insignificant difference in the performance of the VR group in comparison with the control group [10]. In contrast, Rudolphi-Solero et al., who investigated the retention of radiologic anatomy knowledge at the final examinations, and not only after the post-intervention test, found significant superiority of the VR group to the control group [12]. There is a need for more comparative studies to explore to what extent VR can facilitate the retention of radiologic anatomy knowledge. Paech et al. [11], in contrast to Rudolphi-Solero et al. [12] and Lorenzo-Alvarez et al. [10], compared examinations results of students who belonged to different academic years. Again, VR was proved a significantly more effective method than those which were used in the control groups. Of note, this was the only study that comprised a specific comparison of VR with pure radiologic image interpretation training and with anatomy teaching without radiology component. Probably, the three-dimensional (3D) visualization which can be provided by VR may have resulted in better anatomy understanding than conventional 2D imaging. It should be noted that reviews of anatomy education literature have shown that 3D visualization is more effective than 2D one [18,19].

In all the aforementioned three studies, VR-based education was compared with traditional teaching and showed superior effectiveness [10-12]. A possible field of future research is the further comparison of VR with traditional imaging modalities which have been used for combined radiology and anatomy teaching. Although more research, including comparative studies, is needed to confirm the aforementioned positive results, it seems that VR has the potential to provide effective incorporation of radiology into anatomy education.

Students’ Opinions About the Educational Intervention

All articles of the current review evaluated students’ opinions about the use of VR in radiologic anatomy education and showed that the comments were positive. There were two papers that assessed participants’ general perceptions about the educational intervention [12,14]. In the study by Rizvi and Borges [14], there was no unsatisfied student, while Rudolphi-Solero et al., who quantitatively assessed participants’ perceptions about the overall experience, found that the mean grade was very good and almost excellent [12]. All papers of the current review evaluated students’ opinions about the usefulness of VR in radiologic anatomy learning and there was general agreement that this method was very helpful for anatomy understanding. Of note, the study by May et al. was the only one that did not demonstrate positive students’ perceptions immediately after the educational intervention [15]. May et al. showed that the new curriculum, which comprised VR, was not perceived as useful in the first year of its implementation, but students’ opinions were strongly positive in the third year of its implementation [15]. The authors justified this fact, stating that a new curriculum may be a frustrating task and that there is a learning curve for both students and teachers. With the passage of time, as the teachers were getting accustomed to teaching radiological anatomy, thus providing clinical relevance to the anatomy content, students’ perceptions were strongly in favor of the new curriculum. Overall, the positive comments about the educational usefulness of the use of VR in radiologic anatomy education could be justified by the fact that VR provides 3D visualization, thus enabling better anatomy visualization. The game-based delivery of VR may have also contributed to students’ satisfaction [10,12]. The fact that VR was overall perceived as an effective radiologic anatomy teaching tool can stimulate further studies, especially comparative ones, which can clarify which characteristics of VR are the most useful for enabling radiologic anatomy learning.

Limitations

The current review has some limitations. Firstly, the literature search in the aforementioned databases with our keywords, inclusion and exclusion criteria, may have been insufficient to identify all the studies which investigated the results of the use of VR in the incorporation of radiology into anatomy education. However, in our opinion, it is not highly probable that such studies have not been detected. In addition, if the review had included more than seven articles and if the data had enabled us to perform a systematic review with meta-analysis, the role of using VR to teach radiology in conjunction with anatomy would have been more deeply explored. Systematic reviews with meta-analyses in the future will further clarify the role that VR can play in the integration of radiology into anatomy education.

## Conclusions

The incorporation of radiology into anatomy education with the use of VR has been positively commented on by the students and has been found able to enhance radiologic anatomy knowledge. These data may encourage the implementation of VR in the context of integration of radiology into anatomy education. In the modern educational era, it seems that VR can essentially enhance the value of teaching radiology in anatomy curricula and give clinical context to anatomy, thus providing students with a better learning experience.
